# Study protocol of the ASD-Net, the German research consortium for the study of Autism Spectrum Disorder across the lifespan: from a better etiological understanding, through valid diagnosis, to more effective health care

**DOI:** 10.1186/s12888-017-1362-7

**Published:** 2017-06-02

**Authors:** Inge Kamp-Becker, Luise Poustka, Christian Bachmann, Stefan Ehrlich, Falk Hoffmann, Philipp Kanske, Peter Kirsch, Sören Krach, Frieder Michel Paulus, Marcella Rietschel, Stefan Roepke, Veit Roessner, Tanja Schad-Hansjosten, Tania Singer, Sanna Stroth, Stephanie Witt, Anne-Kathrin Wermter

**Affiliations:** 1 Department of Child and Adolescent Psychiatry, Psychosomatics and Psychotherapy, Medical Clinic, Philipps-University Marburg, Marburg, Germany; 2 Department of Child and Adolescent Psychiatry and Psychotherapy, Medical Faculty Mannheim, Central Institute of Mental Health, Heidelberg University, Mannheim, Germany; 3 Department of Child and Adolescent Psychiatry, Medical University of Vienna, Vienna, Austria; 4 Department of Child and Adolescent Psychiatry/Psychotherapy, University Medical Center Göttingen, Göttingen, Germany; 5 Faculty of Medicine, Philipps University Marburg, Marburg, Germany; 6 Department of Child & Adolescent Psychiatry, Medical Faculty of the Technical University Dresden, Dresden, Germany; 7 Division of Psychological and Social Medicine and Developmental Neurosciences, Faculty of Medicine, TU Dresden, Dresden, Germany; 8 Department of Health Services Research, Carl von Ossietzky University Oldenburg, Oldenburg, Germany; 9 Department of Social Neuroscience, Max Planck Institute for Human Cognitive and Brain Sciences, Leipzig, Germany; 10 Department of Clinical Psychology Central Institute of Mental Health, Mannheim, Germany; 11 Department for Psychiatry and Psychotherapy, University Schleswig-Holstein Campus Lübeck, Lübeck, Germany; 12 Department of Genetic Epidemiology in Psychiatry, Central Institute of Mental Health, Mannheim, Germany; 13 Department of Psychiatry, Campus Benjamin Franklin, Charité - Medical Faculty Berlin, Berlin, Germany

**Keywords:** Autism spectrum disorder, Screening, Diagnosis, Therapy, Social competence training, Oxytocin, Health economics, Genetic, ASD-net, German research network for mental disorders

## Abstract

**Background:**

Autism Spectrum Disorder (ASD) is a severe, lifelong neurodevelopmental disorder with early onset that places a heavy burden on affected individuals and their families. Due to the need for highly specialized health, educational and vocational services, ASD is a cost-intensive disorder, and strain on health care systems increases with increasing age of the affected individual.

**Methods:**

The ASD-Net will study Germany’s largest cohort of patients with ASD over the lifespan. By combining methodological expertise from all levels of clinical research, the ASD-Net will follow a translational approach necessary to identify neurobiological pathways of different phenotypes and their appropriate identification and treatment. The work of the ASD-Net will be organized into three clusters concentrating on diagnostics, therapy and health economics. In the diagnostic cluster, data from a large, well-characterized sample (*N* = 2568) will be analyzed to improve the efficiency of diagnostic procedures. Pattern classification methods (machine learning) will be used to identify algorithms for screening purposes. In a second step, the developed algorithm will be tested in an independent sample. In the therapy cluster, we will unravel how an ASD-specific social skills training with concomitant oxytocin administration can modulate behavior through neurobiological pathways. For the first time, we will characterize long-term effects of a social skills training combined with oxytocin treatment on behavioral and neurobiological phenotypes. Also acute effects of oxytocin will be investigated to delineate general and specific effects of additional oxytocin treatment in order to develop biologically plausible models for symptoms and successful therapeutic interventions in ASD. Finally, in the health economics cluster, we will assess service utilization and ASD-related costs in order to identify potential needs and cost savings specifically tailored to Germany. The ASD-Net has been established as part of the German Research Network for Mental Disorders, funded by the BMBF (German Federal Ministry of Education and Research).

**Discussion:**

The highly integrated structure of the ASD-Net guarantees sustained collaboration of clinicians and researchers to alleviate individual distress, harm, and social disability of patients with ASD and reduce costs to the German health care system.

**Trial registration:**

Both clinical trials of the ASD-Net are registered in the German Clinical Trials Register: DRKS00008952 (registered on August 4, 2015) and DRKS00010053 (registered on April 8, 2016).

## Background

The German Federal Ministry of Education and Research is funding a new research network from 2015 to 2019, providing up to 35 million Euros to investigate mental disorders with the aim of devising and developing better diagnostic and therapeutic measures and strategies for the country’s population by means of basic and translational clinical research. Resulting from a competitive call for research proposals entitled “German Research Network for Mental Disorders”, a network of expert consortia from largely university-based research facilities for children, adolescents and/or adults was established. Each consortium will focus its research on one psychiatric disorder, including autism spectrum disorder, anxiety disorders, attention deficit hyperactivity disorder (ADHD), bipolar disorder, depression, schizophrenia and psychotic disorder and substance-related and addictive disorders [1]. Three cross-consortia platform projects will seek to identify shared causes of diseases and develop new diagnostic modalities for this multitude of mental disorders [1]. The present contribution outlines the study protocol for the consortium for Autism Spectrum Disorder, the ASD-Net.

### Autism spectrum disorder (ASD)

ASD is a severe, lifelong and highly cost-intensive neurodevelopmental disorder characterized by impairments in social interaction (e.g. deficits in appropriate eye contact, facial expression, emotion perception, gesture, social and emotional reciprocity) and communication (e.g. echolalia, stereotyped language, reduced reciprocal conversation), as well as restricted and repetitive behavior (e.g. rigid preferences for routines, repetitive motor mannerisms) [2, 3]. For decades, ASD was believed to occur in 4 to 5 per 10,000 children. Nowadays, the prevalence of ASD is estimated at 1% in children and adolescents [4, 5] as well as adults [6], making ASD as common as major psychiatric disorders, e.g. schizophrenia. Although ASD is often considered a childhood disorder, it persists throughout the lifespan [7]. The psychosocial impairment of individuals with ASD is well known [8]. Affected individuals often show severe difficulties in interpersonal relationships and live socially isolated. Since their adaptive behavior, the ability to function independently, seems to fall short of their cognitive capacities, individuals with ASD suffer from considerable impairment in everyday life [9, 10]. Despite good school and/or occupational education, many individuals with ASD are significantly disadvantaged in terms of employment, social relationships, and physical as well as mental health in their later years [11]. As a consequence, they experience frequent job changes and report diminished overall quality of life [12, 13]. Support to facilitate integration into society (e.g. through specialized rehabilitation services) is frequently lacking, and there has been almost no research into ways of developing more effective intervention programs for adolescents and adults with ASD [14, 15]. Besides demonstrating the detrimental impact of ASD on general well-being, recent research shows an increased prevalence of psychiatric comorbidities, e.g. emotional disorders, depression or ADHD [11, 16–21] as well as other medical comorbidities [22, 23]. This may lead to an increased burden upon families living with an ASD affected child [24, 25].

#### Etiological background

In the expression and severity of core and associated ASD symptoms, a considerable heterogeneity is observed [26, 27]. Obvious particularly in the social interaction domain, variability ranges from a near absence of interest in social interaction to rather subtle difficulties in managing complex social interactions that require an understanding of other people’s beliefs, intentions or goals and other cues of social context. Further factors such as developmental trajectories, gender, level of language, cognitive functioning, adaptive behavior, comorbidities etc. contribute to the considerable heterogeneity of the disorder. Although ASD is a strongly genetically determined disorder with a heritability of up to 90% [28–31], the phenotypic variability is paralleled by a notable genetic heterogeneity [for review see: [32–34]. In the past decade, various ASD susceptibility genes and associated biologically coherent functional pathways have been explored [33]. However, many different genetic patterns are also associated with other psychiatric disorders [32, 33, 35, 36]. Therefore, it is of crucial importance to elucidate the link between specific genetic risk variants including epigenetic mechanisms and impaired neural circuitry underlying deficits in the social interaction domain in ASD [33, 37] (e.g. deficits in Theory of Mind, empathy [38–41] and social motivation [42–46]).

#### Diagnosis

Over the past 30 years, the awareness of ASD has increased remarkably, both in public consciousness and in the health professions. This is manifested, for instance, by the large numbers of children, adolescents and specifically adults who present with a suspected diagnosis of ASD, reflecting the increasing demand for diagnosis and treatment by skilled clinicians. Existing screening instruments for autistic symptoms do, in fact, identify individuals with ASD accurately, but fail to discriminate individuals with ASD from those with other psychiatric disorders and complex neurobehavioral profiles (such as ADHD, emotional and personality disorders and others), especially in high-functioning individuals [47–55]. The so-called gold standard clinical tools comprise a standardized interview (Autism Diagnostic Interview Revised, ADI-R), combined with a standardized behavior observation (Autism Diagnostic Observation Schedule-Generic, ADOS-G) and a differential diagnostic examination (requiring up to six hours altogether). Specialized training is needed to become proficient in administering these instruments. Validation studies of these ASD-specific instruments for adults are sparse.

In conclusion, a main issue in health care services is the correct and economical decision on who does or does not require a time- and cost-intensive expert diagnosis for ASD. More trained specialized clinicians and quality management of the diagnostic process are needed to maintain diagnostic quality, improve health services and avoid severe comorbid disorders and socioeconomic burden.

#### Therapy

Although ASD is considered a neurobiological disorder, primary treatments nowadays consist of psychological and educational interventions to address the core deficits related to the disorder. To date, the best empirical evidence exists for early intensive behavioral intervention (EIBI) applying the principles of applied behavior analysis [56]; however, studies on the effectiveness of social skills group training have seen a recent increase, especially for adolescents with average and above-average cognitive skills [57]. Randomized controlled trials (RCTs) using stringent inclusion and exclusion criteria provide evidence that social skills training (SST) is moderately effective in improving social competence and decreasing loneliness in children and adolescents with ASD [58]. Little is known however, about the potentially enhancing effect of additional oxytocin (OXT) treatment along with SST on the acquisition of social competence in ASD. While a growing number of studies show the efficacy of behavior-based interventions in ASD, research on the combination of psychotherapeutic interventions and concomitant pharmacological treatment strategies is still sparse. To date, only a small body of research supports the notion that administration of additional medication may have the potential to enhance effects of psychotherapeutic/behavioral therapies. Since OXT has been identified as a powerful enhancer of neural activity related to social cognition, the formation of social bonds and socially reinforced learning [59–62] it is of particular relevance for ASD and its treatment options. In the last decade, a rapidly growing number of interdisciplinary (pharmacokinetics, (epi)genetics, neuroimaging, imaging genetics, clinical) studies have consistently indicated that OXT plays an important role in modulating human social behavior with translational relevance for understanding ASD [for review see: [63]].

Pioneering but strong evidence suggests that OXT has the potential to enhance motivation and attention to social cues in patients with ASD [62], facilitating the processing of affiliative emotions, social reward and higher cognitive functions such as empathy and Theory of Mind (ToM) in the long term. A recently published review and a meta-analysis [63, 64] summarizing the potentials and limitations of pharmacotherapeutic applications of OXT, came to the conclusion that studies on ASD did show significant effect sizes and that more sophisticated and targeted clinical trials were required. Beyond a therapy augmenting effect, it has been demonstrated that (epi)genetic factors (e.g., methylation differences) influence an individuals’s response to OXT, either by directly acting on OXT genes or via the regulation of genes in pathways related to OXT [65, 66]. Accordingly, genotypic effects are inconsistent [67] and epigenetic factors have to be taken into account to identify those that contribute to the *acute* and *long-term* effects of intranasal OXT.

In summary, cumulative evidence regarding OXT treatment clearly indicates its effectiveness in modulating social behavior and the neural processing of social stimuli in healthy individuals. Several studies on ASD highlight the therapeutic potential of intranasal OXT through effects on core dimensions of social cognition and affected brain systems. However, the exact neural processes, the specificity of this effect for the social domain, and the interaction between different neural networks and neurotransmitter systems still remain to be determined. There is no doubt that a complex disorder such as ASD requires a multifaceted treatment approach. As ASD is a predominantly neurobiologically determined disorder, neurobiological-based approaches, alongside behavior-based methods, should be considered in the treatment of ASD. Moreover, as ASD is a highly heterogeneous disorder, the prediction of treatment response in different subtypes of ASD should be included in well-designed intervention studies in order to correctly allocate individuals to treatment settings [15].

#### Health service utilization and costs

In recent years, the reported prevalence of ASD has increased markedly in Western countries, including Germany [68]. As a consequence, the demand on ASD-specific health services has risen, and there is a need for an improved understanding of adequate diagnostic and therapeutic pathways for these patients. Pathways to an ASD diagnosis are often time-consuming and complicated, with perceived stigma being a potential barrier [69]. Suboptimal pathways to ASD diagnosis can result in dissatisfaction in the carers of patients with ASD, and might preclude timely and adequate treatment of the condition [70]. According to data from the UK and the US, lifetime direct (medical and non-medical) and indirect costs per individual with ASD amount to 1.2–2.4 million USD [71]. The substantial burden of ASD on health and social services has been shown to be greater than that of other childhood illnesses such as diabetes, asthma or intellectual disabilities [72]. ASD begins in childhood, continues across the lifespan, and requires complex and highly specialized health, educational, and vocational services over many years. This burden is compounded by out-of-pocket expenses, e.g. for complementary and alternative medicine (CAM) [73]. In conclusion, ASD is a cost-intensive disorder, with costs increasing with age [74, 75]. Fortunately, these costs can at least be countered by early behavioral interventions: Data from the Netherlands demonstrate long-term savings of approximately € 1.1 million per individual with ASD from early behavioral intervention. Extending these costs to the whole Dutch ASD population, cost savings of € 109–182 billion have been estimated [76]. Nevertheless, German data on ASD-related costs are lacking [77].

### Aims

To address these urgent needs, broad competencies and extensive experience in clinical and research issues are required. Moreover, a comprehensive and well-characterized cohort of patients, diagnosed with standardized procedures, is necessary. The ASD-Net fulfills these prerequisites: it includes the largest ASD cohort in Germany, consisting of patients diagnosed by the gold standard of standardized diagnostic tools (ADOS, ADI-R) including all age and IQ ranges and a significant number of females diagnosed with ASD. The ASD-Net seeks to establish a large clinical and research network focusing on the key challenges in ASD diagnostics, therapy and health economics. The multidisciplinary ASD-Net brings together excellent expertise in ASD, Germany’s largest cohorts in ADI-R−/ADOS-diagnosed children, adolescents, and adults, and state-of-the-art genetic and neurobiological research. The work of the ASD-Net is organized into three clusters concentrating on diagnostics, therapy and health economics. The following research questions and assumptions are examined**: Diagnostic cluster:** Is it possible to develop a reduced number of economical and valid screening items for an early, sensitive and accurate detection of ASD? **Therapy cluster:** What are the *acute* and *long-term* effects of OXT treatment in ASD? Does an adjunctive application of OXT treatment with SST show promise in providing resources to the affected individuals and their families? What are the mediators and moderators of OXT treatment on the level of behavior, neural networks, and (epi)genetics? It is hypothesized that there are long-term synergistic effects of combining psychotherapeutic strategies (SST) with pharmacological treatment (OXT). Furthermore, effects of SST are assumed to be reflected in neurobiological changes in key brain structures associated with ASD in general and with social cognition in particular. These changes are more pronounced in patients receiving a combined treatment with SST and OXT than in patients receiving SST and placebo. Biomarkers are useful for indexing and predicting response to different treatment options in order to assign specific treatment to ASD subgroups. Acute OXT treatment in ASD stimulates neural network activity underlying socio-affective and socio-cognitive processes: OXT normalizes the way in which brain systems process (1) social anxiety and affiliative motivation in ASD, (2) social reward, and (3) emotional empathy as well as Theory of Mind. **Health economics cluster:** What are the medical and non-medical costs of ASD in Germany? Do age, IQ, socioeconomic status or gender impact health and social service utilization and the associated costs? The assessment of service utilization and costs in a very large German ASD cohort helps to draw a naturalistic picture of ASD-related resource utilization and economic consequences, which in turn allows a modeling of potential changes induced by implementing stratified diagnostic and therapeutic interventions.

## Methods

### Diagnostic cluster

Recently, pattern classification methods based on machine learning algorithms have been used to predict or classify individuals of different phenotypes. The idea of machine learning is to find structural patterns in a dataset, and to use these patterns to understand the data and the interrelations of its elements. Thus, the aim of machine learning is to train a computer algorithm to identify complex pattern within a given dataset and to apply the resulting classifier to new individuals to make a better prediction concerning phenotype identification, treatment outcome, and prognosis [78]. The main benefit of pattern classification lies within its potential to detect global, complex, and (in case of ASD) multimodal patterns of abnormalities that can otherwise not be efficiently identified [79]. Training usually takes place in a well-characterized sample by finding an algorithm that best discriminates between classes (ASD vs. non-ASD). Once this algorithm is developed, it can subsequently be used to predict group membership in an independent sample. In order to identify those items of the applied diagnostic tools which show the best discriminatory quality these innovative approaches – already evaluated in the domain of ASD diagnosis in three pilot studies [80–82] – will be applied to the data of 2568 children, adolescents and adults. Retrospective data of the study sample stems from four outpatient specialized ASD clinics in Germany where gold standard diagnostic procedures have been used to confirm the diagnosis of ASD in 1359 individuals. An almost equal number (*N* = 1209) of patients underwent the same procedures, but ASD was ruled out leading to differential diagnoses (e.g. ADHD, language disorder). The outlined machine learning methods (Decision Tree) as well as Support Vector Machine analyses will be used to develop algorithms for screening purposes. For these analyses the software “Konstanz Information Miner (KNIME) 1 Analytics Platform version 3.1.1” is used. The dataset will be divided into training sets and test sets. To avoid overfitting cross-validation will be performed and the dataset will be split into five subsets. Four training sets will be used to build the model. With the test set we will measure the performance of the model. By performing several iterations, this method systematically uses another subset for testing in each iteration. In doing so, the accuracy estimation is computed over multiple test sets. In a second step, the developed algorithm will be tested in a completely independent sample of new-incident individuals with suspected ASD.

On the basis of this large, well-characterized sample, further analyses will be undertaken to improve the efficiency of diagnostic procedures. For this purpose, sub-phenotype identification of ASD will be examined with machine learning techniques. With the help of exploratory and confirmatory factor analysis, the factor structure of algorithm items from the diagnostic instruments will be examined and confirmed in order to identify separable dimensions of the ASD symptomology. To explore the overlap of symptoms of ASD with other disorders, parent-reported and directly observable ASD symptoms will be compared between individuals who did and did not receive a diagnosis of ASD. Group comparisons using analysis of variance (ANOVA) as well as analyses of covariance (ANCOVA, co-varying for age, intelligence) will be undertaken. Thus, we hope to identify diagnostic elements that show both a shifted distribution by diagnostic group and also diagnostic items that differentiate best between ASD and specific non-ASD groups.

Additionally, we will collect and store biomaterial from Germany’s largest cohort of individuals with ASD for intended genetic analyses. In particular, analyses for candidate genes which are known to be involved in the OXT pathway (e.g. *OXTR, LNPEP, AVP, CD38*) will be undertaken.

### Therapy cluster

#### Oxytocin-induced enhancement of SST in ASD (clinical trial)

This randomized, placebo-controlled, double-blind clinical trial aims to test the effectiveness of OXT in enhancing the acquisition of social skills during standardized group-based social skills training (SST) and to test the effectiveness of OXT in maintaining acquired social skills over time. *N* = 168 children and adolescents with ASD aged 8–18 years will be included. Inclusion criteria are: diagnosis of high-functioning autism (F84.0 according to ICD-10), Asperger syndrome (F84.5), atypical autism (F84.1), male patients, and age 12 ≤ years ≤18. Exclusion criteria are: IQ < 75, obsessive-compulsive disorder, psychotic disorder, major depressive episode with suicidal ideation, aggressive behavior interfering with group therapy, any personality disorder, neurological disorder, cardiovascular and endocrinological disorder, hypersensitivity to OXT, other medical disorder interfering with therapy, and group-based SST during the last 6 months prior to study.

**Fig. 1:**
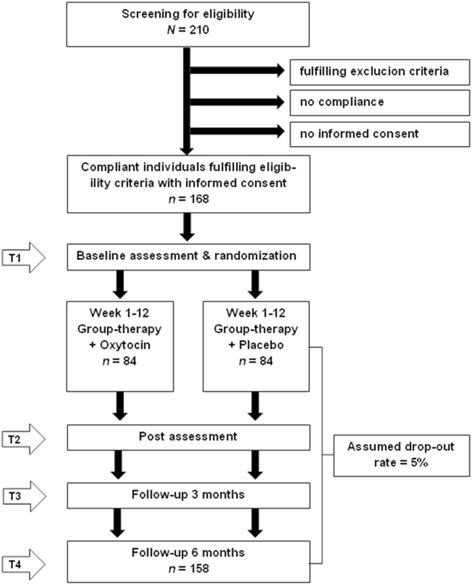
Trial flow. Randomized, placebo-controlled, double-blind clinical trial with 5 phases of examination: T0) Screening; T1) Baseline, 12 weekly SST in combination with OXT vs. 12 weekly SST without OXT; T2) Post-assessment; T3) Follow-up 3 months after end of treatment; T4) follow-up 6 months after end of treatment

Figure 1 describes the design of the trial. In a randomized, placebo-controlled, double-blind clinical trial, participants will receive either OXT or placebo in the form of intranasal spray in addition to group-based SST of 12 weekly sessions based on a manualized group therapy program for ASD [58]. After consent is given, the initial screening will begin in order to clarify inclusion and exclusion criteria, encompassing the DIPS (Diagnostic Interview for Children and Youth for DSM-IV) in order to assess comorbid conditions. This will also comprise saliva samples (cheek swabs) as well as comprehensive medical evaluation including electrocardiogram. After excluding cardiovascular diseases, every ASD patient will receive an intranasal test dose of OXT under the control of a physician (18 IU OXT for age 12–15 or 24 IU for age 16–18). In the case of adverse side effects, patients will be monitored by a physician and excluded from the study. Subsequently, patients with ASD will be randomly assigned to the OXT group or to the placebo group. Group therapists, participants and caregivers will be blind to the participants’ allocation to OXT or placebo. Baseline assessment (T1) will include administration of questionnaires and tests assessing measures of primary and secondary endpoints; demographic variables and saliva samples will also be collected. During the following 12 weeks, group-based SST will be conducted in weekly sessions delivered in combination with OXT or placebo administered 40 min prior to each group-based SST, respectively. SST will be delivered by trained behavioral therapists in groups of 5–6 participants with ASD. Each SST session will be structured and will follow a standard sequence of activities, including introduction of a specific skill, modeling of the skill, role playing with rehearsal/ practice of the modeled skill, discussion, and individualized performance feedback. Common topics will include emotion recognition and regulation, social competence, social problem solving, and social communication. Post-assessment (T2) will include identical measures to at baseline (T1). Follow-up 1 (T3, 3 months after the end of treatment) and follow-up 2 (T4, 6 months after the end of treatment) will again assess the primary and secondary outcome measures. Primary efficacy endpoints are changes in the total raw score of the Social Responsiveness Scale (SRS) [83] rated by parents between baseline assessment (T1), post-assessment (T2), follow-up 3 months after end of intervention (T3) and follow-up 6 months after end of intervention (T4). Secondary endpoints are changes in the total raw score of: (1) prosocial behavior and peer relationship problems (SDQ) [84], (2) empathy (Multifaceted Empathy Test, MET-J) [85], (3) depression (DIKJ) [86], (4) psychological distress (SSKJ) [87], (5) physiological distress (cortisol levels), and (6) quality of life (CHIP-CE) [88].

An effect for OXT vs. placebo of d = 0.60 was calculated based on studies which included patients with ASD. Since in the present study, an additional SST is included for both groups, a more conservative effect of *d* = 0.45 is assumed as realistic and clinically relevant. Using a two-sample t-test with a two-sided significance level of 5%, a total of 158 patients is required for the analysis to achieve a power of 80% (nQuery Advisor 7.0). Due to an expected drop-out rate of 5% and assuming that 20% of screened patients are not eligible, *N* = 210 patients will be assessed for eligibility, of whom *n* = 168 patients will be allocated to either the OXT or placebo group providing at least the desired power of 80%.

A confirmatory analysis of the primary efficacy endpoint will be conducted. Analysis of covariance (ANCOVA) will be applied including baseline SRS total raw score, age and IQ as continuous covariates and center, comorbidity and medication status as factors for control. Three primary endpoints are of interest and hence three null hypotheses are tested. To ensure a multiple type I error rate of 5%, a hierarchical test procedure will be applied: The first null hypothesis states that the change in the total raw score of the SRS between T1 and T2 is equal for both groups (H01: μT = μC) and is tested at a two-sided significance level of 5% against the alternative hypothesis (H01: μT ≠ μC). If the first null hypothesis can be rejected, the second null hypothesis for the change in the SRS total raw score between T1 and T3 will be tested again at a significance level of 5%. Finally, if the second null hypothesis can be rejected, the third null hypothesis for the change in the SRS total raw score between T1 and T4 will be tested at a significance level of 5%. All secondary outcomes including safety data will be evaluated descriptively, using appropriate statistical methods based on the underlying distribution of the data. Descriptive *p*-values are reported together with 95% confidence intervals for the corresponding effects. Missing values concerning primary outcomes are dealt with by application of the mixed-effects model for repeated measures, which turned out to show favourable characteristics in terms of type I error rate, power, and bias of estimates as compared to alternative methods dealing with missing values, such as last-observation-carried-forward (LOCF). Missing values for the covariates will be replaced by multiple imputations.

This clinical trial will be conducted and analyzed in accordance with ICH-GCP guidelines, the Declaration of Helsinki, German Drug Law (AMG) and Data Protection Law. The trial including consent procedures been approved by the leading ethics committee of Heidelberg University and by the Federal higher authority (Federal Institute for Drugs and Medical Devices, BfArM). All Parents/caregivers gave written informed consent, minor participants gave assent for participation in the study. It is registered in the German Clinical Trials Register (DRKS00008952).

#### Neurobiological markers for SST response in ASD

This study is a supplement to the study outlined above and is based on the assumption that effects of an OXT-enhanced SST should be particularly observable in brain regions associated with ASD and OXT like the social brain (temporoparietal junction, temporal pole, precuneus and medial prefrontal cortex), reward circuits and the amygdala [89]. We will therefore apply a battery of three experimental fMRI tasks to examine the effect of OXT administration on neural activation in *N* = 100 patients involved in the above-mentioned clinical trial before and after SST. We will focus on the aforementioned brain regions and use fMRI paradigms which target these regions and functions. Moreover, we will delineate OXT-specific modulation of the social brain using a Theory of Mind (ToM) task to activate the mentalizing network [90], an affective matching task focusing particularly on the amygdala [91], and an adapted version of a validated reward task combining both social and non-social cues as well as social and non-social rewards [92]. MR sequence protocols and stimulus presentation settings have been harmonized across the two participating sites. The main outcome variables will be neural activity in and connectivity between the aforementioned brain regions of interest during the three tasks. Behavioral data such as accuracy ratings and response times will also be used. We will identify specific neurobiological mechanisms associated with therapy response as well as particular neurobiological signatures before treatment that are associated with treatment response. Results should further allow us to develop hypotheses regarding how to tailor treatment to different subtypes of ASD and to correctly allocate individuals to treatment settings.

We assume an effect size of d = .45. Given a sample size of 90 (100 minus 10% drop out), we have 85% power to detect a difference (pair-wise t-test) at a significance level of *p* < .001, which is a threshold often used for whole brain fMRI studies.

Preprocessing and analysis of functional and structural images will be processed using SPM12 toolbox (http://www.fil.ion.ucl.ac.uk/spm/) within the Nipype framework (http://nipy.org/nipype/). The quality of the fMRI data will be ensured by manual inspection, using artifact detection tools (ART) and a stringent motion control procedure. To account for group-specific structural brain difference a specific DARTEL template will be created [93]. The slice time corrected functional data will be realigned, registered, and normalized to MNI using the DARTEL template. Whole brain data (or extracted summary metrics) will be compared using linear mixed effects (LME) models with random intercepts, treatment as between- and timepoint as the within-subject factor. We will covary for age and other possibly confounding variables.

#### Modulatory effects of acute OXT treatment in ASD (clinical trial)

This clinical study is a randomized, double-blind, cross-over, placebo-controlled, multicenter functional magnetic resonance imaging study with two arms [94]. The aim is to characterize the acute effects of OXT on neural network activity during socio-affective and socio-cognitive functioning in ASD and to compare these to healthy controls (HC). A sample of 102 male ASD patients (age 19 ≤ years ≤40) diagnosed with Childhood Autism (F84.0 according to ICD-10), Asperger syndrome (F84.5 according to ICD-10), or atypical autism (F84.1 according to ICD-10) will be recruited. These will be matched (IQ and age) with healthy control participants (*N* = 66). Both groups will receive OXT and placebo nasal spray on two different days at an interval of two weeks to ensure a sufficient wash-out time after OXT treatment. Both ASD patients and healthy control participants will be randomized to determine whether they receive OXT on the first or the second visit. Investigators and participants will be blind to the study condition. Exclusion criteria are: IQ < 70; traumatic lesions of the brain; serious neurological diseases (e.g. epilepsy); contraindications for OXT administration (e.g. known metabolic or endocrinological disorders; cardiac disorders; known hypersensitivity to nasal sprays or other drugs); contraindications for the MRI assessment (e.g. incorporated metal, agoraphobia); comorbid drug or alcohol abuse or dependence. In two consecutive sessions, ASD patients and HC will receive 24 IU of OXT or placebo as intranasal spray 45 min prior to the fMRI assessment of activity in neural networks associated with social processes. MRI assessment will last for 60 min and will encompass three experimental paradigms that probe neural activation in social brain systems that have shown altered activity in ASD in previous studies (emotional matching [adapted from: [91]], social orienting, social reward anticipation and consumption [adapted from: [95, 96]], empathy, compassion and Theory of Mind [adapted from: [97, 98]]), a resting state and structural scans.

The primary outcome will be neural network activity, measured with functional magnetic resonance imaging while participants perform socio-affective and socio-cognitive tasks. Secondary outcome measures will consist of behavioral and physiological measures respectively which comprise accuracy ratings and response times in the conducted tasks (e.g. Theory of Mind task) as well as skin conductance. Complementing performance, descriptive measures of trait alexithymia, interpersonal reactivity and social anxiety will be evaluated. Additionally, the effect of OXT receptor gene variants its potential influence on the primary and secondary outcome measures will be analyzed. Effect size of d = 0.65 will be detected with 80% power and a significance level of *p* = 0.001 (corrected) with a total sample size of 88 patients with ASD. With a drop-out estimation of 13%, *N* = 102 patients have to be recruited as well as a matched control group of healthy controls *N* = 88).

All fMRI data will be pre-processed and analyzed in the statistical parametric mapping framework (SPM, www.fil.ion.ucl.ac.uk/spm). Standard routines and templates will be used for the fMRI data analysis and pre-processing. BOLD activation will be analyzed with a repeated measures analysis of variance (ANOVA) to compare the effects of OXT treatment to placebo in the ASD group. An independent samples t-test will be conducted to compare neural network activity of ASD patients to those of the HC group under placebo as reference. Further, to characterize OXT effects in the HC group and test for different treatment responses in ASD patients and HCs, repeated measures ANOVA will be conducted. To control for the increased type 1 error in the analysis of the imaging data, corrections for multiple-comparisons will be applied as implemented in SPM based on the estimated smoothness of the statistical map using Gaussian random-field theory, and the T and F maps will be thresholded accordingly. Thresholding of the imaging data will be conducted in two consecutive steps, first as an exploratory analysis within the whole brain, and second within the predefined regions of interest.

This clinical trial will be conducted in accordance with ICH-GCP guidelines and the Declaration of Helsinki. The trial has been approved by the leading ethics committee of Lübeck University, the concomitant ethical board of Leipzig and the Federal higher authority (Federal Institute for Drugs and Medical Devices, BfArM). Prior to testing, participants’ written informed consent will be acquired. Consent procedures have been approved by the leading ethics committee. The trial is registered in the German Clinical Trials Register (DRKS00010053).

#### (Epi)genetics

Little is known about the molecular mechanisms underlying the impact of OXT on a behavioral and/or neural level. In view of the individual variability in OXT response, the consideration of individual factors such as gender and genetic and epigenetic variations is warranted in studies investigating the efficacy of OXT administration in ASD treatment [65]. We will collect and store biomaterial including pre−/post-treatment biomaterial information of participants involved in the above-mentioned clinical trials. In this way, we aim to identify genetic and epigenetic factors that are a) predictive at baseline for the patients’ treatment [OXT, social skills training (SST) + placebo, SST + OXT] outcome and b) associated with the response to *acute* and *long-term* OXT administration. By identifying implicated genetic factors and methylation changes, we will c) gain new insights into the molecular mechanisms underlying ASD and the OXT response. Data at baseline and after treatment will be analyzed not only with respect to the categorical diagnoses but also regarding behavioral and neuroimaging sub-phenotypes.

### Health economics cluster

*WP1 Cost-of-illness study.* Using the Client Service Receipt Inventory (CSRI), we will collect data on service utilization in a large sample of ASD patients (*N* = 1419) in order to assess direct and indirect ASD-related costs. An extensive literature review will be carried out to gather information on services used and costs of each service unit (e.g. CAM, special education lessons). Based on these data, we will calculate annual and lifetime ASD-related costs from a societal perspective (including, e.g., education costs and parental productivity loss), using a micro-costing approach, stratified by age, gender, and IQ. *WP2 Decision-analytic model* **:** We will perform an extensive literature review, with a focus on therapy results and consistency of therapy outcomes in the addressed age group. On this basis, a decision-analytic Markov model will be formed [78], simulating early therapeutic interventions before vs. after five years of age in ASD patients. Using a cost-benefit approach, potential short-term and long-term cost effects will then be calculated. *WP3 Health services utilization pathway:* Using data from the total sample as well as a sub-sample of patients from WP1 (new-incident ASD patients), we will assess diagnostic pathways and barriers to service utilization in patients with a first-time ASD diagnosis. Based on these data, we aim to identify subgroups of patients with differential needs, and suggest stratified diagnostic and therapeutic pathways, with the objective of more efficient resource utilization. Age and gender aspects, socioeconomic status and ASD-related stigma will be addressed in all work packages, as these factors potentially influence both diagnostic pathways and subsequent service utilization.

Standardised data collection forms will be used and data will be entered in a Case Report Forms (CRF) created in OpenClinica®. Mainly descriptive statistics will be used for analyses on health services use. Prevalences (e.g. on use of CAM use) alongside with 95% confidence intervals will be calculated for dichotomous variables and means (with standard deviation) or median (with interquartile range) will be presented for continuous variables (e.g. number of hospital days). Unadjusted individual total costs will be calculated for each participant by summing up costs of all categories. Relative differences in mean costs between groups (e.g. between different age groups) will be assessed by using a gamma-regression with log-link.

## Discussion

In the present paper, we have identified a number of urgent research questions in different areas of ASD research. The ASD-net will address these questions in the area of diagnosis, therapy and health economics and thereby provide new insights that should improve early diagnosis and treatment of the disorder and help to economically optimize their application. However, in the following section, we also wish to discuss some critical aspects that we need to take into account when conducting our research program.

### Diagnostic cluster

Machine learning is deemed to have great potential to enhance diagnostic and interventional research and to be especially useful in investigations involving the relatively prevalent and heterogeneous syndrome of ASD [99]. However, there are relevant critical aspects to be considered when applying machine learning techniques within the diagnostic process of ASD. Wall and colleagues [82] used machine learning to shorten the behavioral observation procedure (ADOS) by applying machine learning techniques to automatically identify the fewest number of items for an abbreviated algorithm. In response, Bone et al. [99] argued that ADOS codes might not be valid outside the context of a full ADOS administration. In the light of the fact that administration time was not actually reduced with the approach proposed by Wall et al. [82], the proposed procedure seemed to be without benefit. Particularly as the input data to the machine learning algorithms, i.e. the ADOS codes are to be considered reliable and valid only when educed by a specialist administrator using standardized materials in the ADOS context, a semi-structured social process. Furthermore, it was argued that the established algorithms were not validated on independent data and an adequate sample [80, 99]. It seems prudent to use larger and more balanced datasets, a more detailed characterization of core ASD components from multiple perspectives, and cross-validation in an independent sample. Within the ASD-Net, these crucial aspects can be resolved: In our large, well-characterized sample, individual differences concerning age groups or other sub-phenotypes can be detected and an evaluation in an independent and clinical relevant sample is possible. Given these circumstances, the aims of the diagnostic cluster to develop and evaluate a screening instrument for the early detection of ASD across all age ranges, IQ ranges, and gender seems feasible. This will facilitate early and effective access for affected individuals to specialized procedures and therapeutic interventions. Moreover, economical screening instruments will reduce the increasing amount of health care utilization in terms of time- and personnel-intensive diagnostic investigations and ultimately reduce the rate of false-positive and false-negative cases. In turn, this will improve the efficiency of diagnostic procedures over the lifespan and consequently enhance the effectiveness of the health care system.

### Therapy cluster

Several studies have documented that intranasal OXT shows promising effects: reduction of social fear and stress and increase in trust, emotion recognition, Theory of Mind, empathy and bonding behavior. Although clinical trials of intranasal administration of OXT for treating psychiatric problems have yielded mixed results [61, 100–102], most authors conclude that intranasal administration of OXT is a potentially useful intervention for the treatment of ASD [62, 63, 103]. Notably, a recently published meta-analysis [64] summarized recent studies on pharmacotherapeutic applications of OXT treatment in order to explore its potential and limitations. It concluded that studies on ASD showed significant effect sizes (*d* = 0.57; *N* = 68; 95% CI: 0.15–0.99; *p* < 0.01). As there is no effective medical treatment for the core ASD symptoms, and psychological treatments remain costly, time-intensive and developmentally sensitive in terms of efficacy, OXT-based therapies may have the potential to close this gap. However, more studies are needed that determine the best treatment target and identify the underlying mechanisms of behavioral change.

Evidence shows that acute OXT administration is associated with changes in numerous markers critical to the functioning of the brain circuitry underlying social deficits in ASD [adapted from: [102, 104–106]], even though the neural processes and the specificity of OXT effects on socio-affective and socio-cognitive functioning are not fully understood [59, 64, 101, 107, 108]. Although recent evidence seems to suggest that OXT might optimize neural transmission in socio-affective and socio-cognitive brain circuits and enhance reward, motivation, and learning to improve therapeutic outcomes, the current evidence regarding the therapeutic benefit from extended OXT treatment remains very limited.

The very first studies investigating the efficacy, tolerability and safety of extended OXT treatment in individuals with ASD produced mixed results: An Australian study [109] examined the effect of OXT and placebo nasal spray (24 IU per day) for 5 weeks and found that OXT led to significant improvements in caregiver-rated social responsiveness. In adults (*N* = 19), no significant changes were observed in the primary outcome measures (social function/cognition and repetitive behaviors; results suggested improvements after 6 weeks on measures of social cognition). Two other studies found no effect in youth with ASD [110], or of the long-term administration of intranasal OXT in adolescent and adult ASD subjects with intellectual disability (*N* = 29) [111]. To date, only one pilot study has combined the administration of OXT with behavioral treatment: 38 male youths (7–16 years old) with ASD received intranasal OXT or placebo once daily over four consecutive days during parent-child interaction training sessions [112]. This very short intervention did not significantly improve emotion recognition, social interaction skills, or general behavioral adjustment. As the safety of OXT is reported to be very good [113], but information on efficacy, especially in children and adolescents with ASD, is still limited and ambiguous, more elaborated clinical trials with OXT are warranted [63], and the proposed study protocol has the potential to address this need.

In summary, the aim of the therapy cluster is to unravel whether and how combined behavioral and pharmacological treatments modulate behavior through neurobiological pathways in ASD. This is of central importance for developing biologically plausible models for the symptoms in the social domain and successful future therapeutic interventions in ASD. In addition, the acute effects of OXT administration on the neurobiology of social cognition will be tested. This will serve as a basis from which to further disentangle the acute and long-term effects of OXT on neurobiological pathways. The determination of characteristic biomarkers for (sub-) phenotypes by integration of (epi)genetic, behavioral and neuroimaging data may also help to predict treatment response early and allocate patients to adequate treatments in order to minimize personal distress and financial resources. One potential challenge lies in the recruitment of the required large sample of adolescents. These have to be willing to participate in the study, to take the time (besides, e.g., school, leisure activities, special education) to attend at the same time slots as other group members, have no (metal) retainers or other dental braces, live near to the study centers or have parents who can drive them to the study center once a week.

### Health economics cluster

The substantial burden of ASD on the public sector has been documented for the USA and the UK. However, data on ASD-related costs in Germany are lacking. Thus, the main goal of the health economics cluster is to assess the status quo in terms of resource allocation and costs for ASD in Germany. This will help to identify subgroups of ASD patients with differential needs, enable stratified diagnostic and therapeutic pathways for ASD patients, and facilitate cost-saving resource allocation. Together, the results of this cluster have significant potential to improve health service utilization experiences for patients with ASD and their caregivers, and to optimize resource utilization in these patients.

## Conclusions

In sum, the highly integrated structure of the ASD-Net guarantees sustained collaboration of clinicians and researchers to reduce individual distress, harm, and social disability of patients and costs for the German health care system. In light of the enormous burden ASD represents for concerned individuals, families and society as a whole, a sustainable improvement in the financial support for those researching ASD is absolutely essential. However, the greatest challenge will be the allotted duration for the ASD-Net, which seems to be a rather tight schedule. The resources concerning personnel and time are restricted, and an extension to continue after the funded time period and extension of funding will most likely be necessary.

## Abbreviations

ADHD: attention-deficit/ hyperactivity disorder
ADI-R: Autism Diagnostic Interview-Revised
ADOS: Autism Diagnostic Observation Schedule
ASD: Autism Spectrum Disorder
AVP: arginine vasopressin
BfArM: Bundesinstitut für Arzneimittel und Medizinprodukte
BMBF: Bundesministerium für Bildung und Forschung
CHIP-CE: Child Health and Illness Profile–Child Edition
DIKJ: Depression Inventory for children
fMRI: Functional magnetic resonance imaging
IQ: Intelligence Quotient
IU: International Units
LNPEP: leucyl and cystinyl aminopeptidase
MET-J: Multifaceted Empathy Test
OXT: Oxytocin
OXTR: Oxytocin receptor
RCT: Randomized controlled trial
SDQ: Strengths and Difficulties Questionnaire
SSKJ: Stress and stress coping inventory
SST: social skills training
WP: Work Package
